# Using the Honey Bee (*Apis mellifera* L.) Cell Line AmE‐711 to Evaluate Insecticide Toxicity

**DOI:** 10.1002/etc.5500

**Published:** 2022-12-09

**Authors:** Michael Goblirsch, John J. Adamczyk

**Affiliations:** ^1^ Thad Cochran Southern Horticultural Laboratory, Agricultural Research Service US Department of Agriculture Poplarville Mississippi USA

**Keywords:** *l(2)efl 410087a*, cytotoxicity, honey bee cell culture, nontarget organism, heat shock proteins, sublethal doses

## Abstract

One of the main contributors to poor productivity and elevated mortality of honey bee colonies globally is insecticide exposure. Whole‐organism and colony‐level studies have demonstrated the effects of insecticides on many aspects of honey bee biology and have also shown their interactions with pathogens. However, there is a need for in vitro studies using cell lines to provide greater illumination of the effects of insecticides on honey bee cellular and molecular processes. We used a continuous cell line established from honey bee embryonic tissues (AmE‐711) in assays that enabled assessment of cell viability in response to insecticide exposure. We exposed AmE‐711 cells to four formulations, each containing a different insecticide. Treatment of cells with the insecticides resulted in a concentration‐dependent reduction in viability after a 24‐h exposure, whereas long‐term exposure (120 h) to sublethal concentrations had limited effects on viability. The 24‐h exposure data allowed us to predict the half‐maximal lethal concentration (LC50) for each insecticide using a four‐parameter logistical model. We then exposed cells for 12 h to the predicted LC50 and observed changes in morphology that would indicate stress and death. Reverse transcription–quantitative polymerase chain reaction analysis corroborated changes in morphology: expression of a cellular stress response gene, *410087a*, increased after an 18‐h exposure to the predicted LC50. Demonstration of the effects of insecticides through use of AmE‐711 provides a foundation for additional research addressing issues specific to honey bee toxicology and complements whole‐organism and colony‐level approaches. Moreover, advances in the use of AmE‐711 in high‐throughput screening and in‐depth analysis of cell regulatory networks will promote the discovery of novel control agents with decreased negative impacts on honey bees. *Environ Toxicol Chem* 2023;42:88–99. Published 2022. This article is a U.S. Government work and is in the public domain in the USA. *Environmental Toxicology and Chemistry* published by Wiley Periodicals LLC on behalf of SETAC.

## INTRODUCTION

Insecticides are a contributing factor to poor honey bee performance and elevated rates of colony death in the United States and other parts of the world (Goulson et al., 2015). Honey bees are not the targets of insecticide applications, but they may be exposed when foraging in and around treated landscapes (Krupke et al., 2012). Treated landscapes may serve as a point of contact for honey bees to become exposed to insecticides; however, beeswax comb, food stores, and nestmates within the hive may also become contaminated with insecticide residues (Mullin et al., 2010). This scenario of widespread exposure could encompass a bee's entire life cycle. Regardless of the route of exposure, the ubiquitous presence of insecticides in the environment and in colonies warrants further investigation into their effects on honey bee health.

A growing body of research has demonstrated acute and sublethal effects of individual and mixtures of insecticides at many levels of honey bee biology. Studies have shown negative effects on immature and adult survival (Dai et al., 2017; Wade et al., 2019); learning and behavior (Aliouane et al., 2009; Decourtye et al., 2004); queen bee health (Fine, 2020; Wu‐Smart & Spivak, 2016); and interactions with pathogens (Di Prisco et al., 2013; Doublet et al., 2015). Undoubtedly, these and numerous other studies that use whole‐organism or colony‐level experimental designs fill gaps in our knowledge about the impacts that insecticides have on honey bees with potential ramifications for beekeeping and environmental sustainability. However, when exploring the effects that insecticides have on honey bee cellular and molecular processes, whole‐bee and colony‐level approaches, whether conducted in the field or laboratory, may present limitations for providing a high level of control against confounding factors. Differences in the level of uniformity between experimental units, including history of chemical exposure, can be a source of unwanted variation. Opportunities exist to improve measurement precision at the cell level as it relates to honey bee toxicology. Cultured honey bee cells, which are maintained under highly controlled conditions and are devoid of the complexities of whole‐organism, even superorganism, life may provide a means to better study how insecticides contribute to cellular dysfunction and toxicity.

As in other areas of honey bee biology and disease (Guo et al., 2020), there is a lack of in vitro systems established from explanted honey bee tissues and cells to make direct observations on the cellular responses to abiotic and biotic stressors. Cell culture offers a platform from which a population of host cells can be maintained artificially in a simplified, regulated environment. Equal numbers of cells can be distributed across replicate units (e.g., multiwell plates), which can then be manipulated by addition or subtraction of variables to the culture environment. The outcome of these manipulations can be observed and quantified via complementary techniques, such as high‐resolution imaging, bioassays, and bioinformatics to draw comparisons with a healthy, control phenotype. Insecticides are one variable that can be added to a cell culture system to establish a toxicity profile at the cellular level with the potential for extrapolation to higher levels of biological organization. At a time when there is rapid development of new insecticides, cell lines and other nonlethal, sustainable approaches have become attractive alternatives to conventional models that have cost or ethical considerations (Fischer et al., 2019; Haas & Nauen, 2021; Kroglund et al., 2022).

Nearly 1000 continuous cell lines have been established specifically from insect tissues (Bairoch, 2018). Insect cell lines have proved useful for evaluating the cytotoxicity of insecticides (Aljabr et al., 2014). Moreover, recombinant expression of metabolism genes in insect cells have helped shed light on insecticide detoxification mechanisms (Haas & Nauen, 2021; Meng et al., 2020). Notably, some studies have used primary cultures of neuronal cells from bumblebees (Moffat et al., 2015; Moffat et al., 2016; Wilson et al., 2013) and hemocytes from both bumblebees and honey bees (Walderdorff et al., 2018) to assess the neurotoxic and immune responses of insecticides, respectively. Nevertheless, the under‐representation of cell culture systems from bee species in general, and honey bees in particular prompted us to use the honey bee cell line AmE‐711 to demonstrate the effects of insecticides on cell viability, morphology, and cell stress.

The cell line AmE‐711 is a continuous line that was established from embryonic tissues of the western honey bee, *Apis mellifera* (L.) (Goblirsch et al., 2013). We exposed AmE‐711 cells to a dilution series of four formulations, each containing a different insecticide. The insecticides we tested included two acetylcholinesterase inhibitors, the carbamate carbaryl, and the organophosphate acephate; a nicotinic acetylcholine receptor agonist, the neonicotinoid imidacloprid; and a sodium channel modulator, the pyrethroid λ‐cyhalothrin (Insecticide Resistance Action Committee, 2022). The formulations containing these insecticides are commonly used for the control of landscape and ornamental pests; therefore they simulate real‐world applications, and the active ingredients are widely applied via other formulations to agricultural systems in the United States, including crops visited by honey bees (US Geological Survey, 2021). We used fluorimetry to measure the acute (24‐h exposure) and long‐term (120‐h exposure) responses of AmE‐711 cell viability to insecticide exposure. Viability data from the acute exposure experiments were then used to predict the half‐maximal lethal concentration (LC50) of each insecticide. The predicted LC50 concentrations were then applied to AmE‐711 cells to qualitatively observe changes in cell morphology and quantify relative expression of a cellular stress response gene. Our overall goal was to demonstrate the utility of AmE‐711 as a tool in cytotoxicity assays and provide a foundation for further research on the use of the cell line for honey bee toxicology.

## MATERIALS AND METHODS

### Culture of AmE‐711 cells

**Figure 1:**
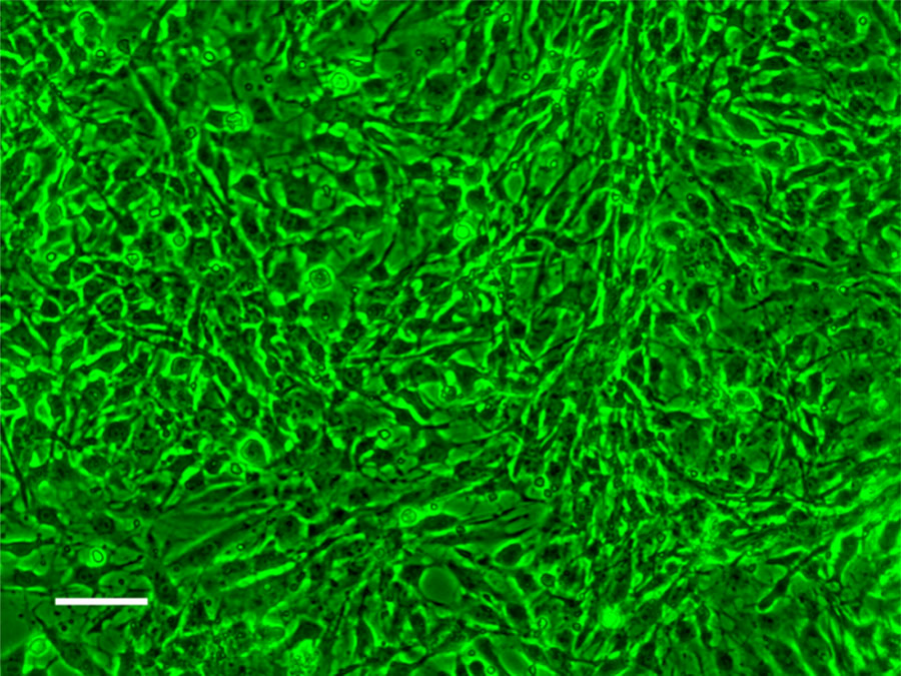
A confluent layer of AmE‐711 honey bee cells in culture. The photomicrograph was taken at 200× magnification using phase contrast microscopy with a green filter. The image was acquired in July 2021 and was adjusted for input levels. The AmE‐711 cells are mainly spindle‐shaped with the branched cytoplasm typical of a fibroblast‐type morphology. The cells are strongly adherent. Scale bar = 50 µm.

The AmE‐711 cell line was obtained, with permission, from the University of Minnesota (Minneapolis, MN, USA). The cell line was maintained using Schneider's Insect Medium containing L‐glutamine and sodium bicarbonate. The base medium was supplemented with 10% heat‐inactivated fetal bovine serum (FBS), both from Millipore Sigma. The cell line AmE‐711 is persistently infected with the honey bee positive‐sense, single‐stranded RNA virus, Deformed wing virus (Carrillo‐Tripp et al., 2016), a common pathogen of honey bee colonies. The virus does not cause an overt cytopathology in currently maintained AmE‐711 cultures, and the phenotype of the line remains unchanged from its pre‐2015 characterization (Goblirsch et al., 2013). The phenotype of AmE‐711 cells can be characterized as strongly adherent, fibroblast‐type cells that are not contact inhibited (Figure 1).

The AmE‐711 cells were cultured in Falcon nonvented 75‐cm^2^ cell culture treated flasks (Corning) in a nonhumidified incubator set at 32 °C. Healthy, confluent cells were detached from the flask substrate by removing the culture medium and incubating the cell layer in the presence of 0.25% trypsin‐ethylenediaminetetraacetic acid (Thermo Fisher Scientific) for up to 10 min at 32 °C. Some flasks were washed with sterile 1× Dulbecco's phosphate‐buffered saline (dPBS), modified without calcium chloride and magnesium chloride (Millipore Sigma) prior to the addition of trypsin to facilitate detachment. Trypsinization was stopped by adding fresh growth medium containing 10% FBS, and detached cells were pooled from several flasks with a similar passage history and mixed thoroughly before seeding in multiwell plates.

### Chemicals—Insecticide formulations

The formulations we used were chosen partly because their active ingredients represent four different groups/subgroups of insecticides (Table 1). The insecticides tested were the pyrethroid λ‐cyhalothrin—1α(S*),3α(Z)]‐(±)‐cyano‐(3‐phenyoxyphenyl)methyl‐3‐(2‐chloro‐3,3,3‐trifluoro‐1‐ propenyl)‐2,2‐dimethylcyclopropanecarboxylate contained in Cyonara 9.7 (Control Solutions); the neonicotinoid imidacloprid—1‐[(6‐chloro‐3‐pyridinyl)methyl]‐N‐nitro‐2‐imidazolidinimine contained in Dominion 2 L (Control Solutions); the organophosphate acephate—N‐[methoxy(methylsulfanyl)phosphoryl]acetamide contained in Ortho Systemic Insect Killer Concentrate (Scotts); and the carbamate carbaryl—1‐naphthyl N‐methylcarbamate contained in Sevin Concentrate Bug Killer (Garden Tech). The insecticides were stored in the dark at ambient temperature in a chemical storage facility. The insecticides were thoroughly mixed by manual agitation of the original stock bottles for 1 min prior to taking subsamples to make the working solutions. Working solutions were prepared in Schneider's Insect Medium without FBS but with the addition of 100 U ml^−1^ penicillin, 100 mg ml^−1^ streptomycin (Thermo Fisher Scientific). The concentration of active ingredient provided on the label of each formulation was assumed to be % w/w (US Environmental Protection Agency, 2012) and was 9.7% λ‐cyhalothrin for Cyonara 9.7, 21.4% imidacloprid for Dominion 2 L, 8.0% acephate for Ortho Systemic Insect Killer Concentrate, and 22.5% carbaryl for Sevin Concentrate Bug Killer. The formulations were used “as is” and not submitted for further chemical analysis. Hereafter, the insecticides are referred to by their formulation names unless pairing the active ingredient and formulation names together aided presentation of the results and conclusions.

**Table 1:** Insecticide formulations used in the present study

Formulation	AI	CAS no.	AI %^a^	IRAC group	Mode of action	AI ppm^b^
Cynoara 9.7	λ‐cyhalothrin	91465‐08‐6	9.7	Pyrethroid—3	Sodium channel modulator	1.29–12 901
Dominion 2 L	Imidacloprid	13826‐41‐3	21.4	Neonicotinoid—4A	Nicotinic acetylcholine receptor agonist	3.30–32 956
Ortho Systemic Insect Killer	Acephate	30560‐19‐1	8.0	Organophosphate—1B	Acetylcholinesterase inhibitor	1.08–10 800
Sevin Concentrate Bug Killer	Carbaryl	63‐25‐2	22.5	Carbamate—1A	Acetylcholinesterase inhibitor	2.70–27 000

^a^ Concentration of AI as listed on the formulation label and assumed to be % w/w.

^b^ Estimated range of AI concentrations used in the present study. The specific gravity of the AI was used to transform % w/w to % w/v. The % w/v was then converted to parts per million (ppm).

AI = active ingredient; IRAC = Insecticide Resistance Action Committee.

### Resazurin‐based viability assay

To assess the response of AmE‐711 cell viability to acute and long‐term insecticide exposure, assays were conducted using alamarBlue HS Cell Viability Reagent (ThermoFisher Scientific). The alamarBlue reagent consists of a phenoxazine dye, resazurin (7‐hydroxy‐3H‐phenoxazin‐3‐one 10‐oxide), that is not toxic to cells. When added to cultures, the dye is irreversibly reduced to a pink product, resorufin (7‐hydroxy‐3H‐phenoxazin‐3‐one). The resorufin product is highly fluorescent in living cells, and this signal was detected and quantified using an excitation wavelength of 560/30 nm and an emission wavelength of 590/30 nm per the manufacturer's recommendations.

The AmE‐711 cells were seeded at an approximate density of 1.0 × 10^5^ cells/well using Nunclon Δ surface‐treated 96‐well plates (ThermoFisher Scientific). The volume of culture medium in each well was 100 μl, and the plates were incubated at 32 °C until use. The cells were allowed to recover from the subculture process and adapt to the microplate environment for 2 days prior to insecticide exposure. At the time of exposure (day 3 after seeding), the culture medium was removed and replaced with medium without FBS but containing antibiotics and one of a dilution series of each insecticide. The dilution series for each insecticide was made to achieve a final concentration of the formulation at 0.0 (untreated control), 0.01, 0.05, 0.1, 0.5, 1.0, 5.0, and 10.0% (v/v) for acute exposure and 0.0, 0.001, and 0.01% for long‐term exposure in 100 μl total volume for each well. The range in concentrations of active ingredient that the cells were exposed to was calculated from the label for each insecticide. The purity of the active ingredient was assumed to be 100%. The label concentration of active ingredient (% w/w) was transformed to % w/v using the specific gravity of the active ingredient before converting it to parts per million (ppm) for each dilution (Supporting Information, Table 1). The range in concentrations of active ingredient was 1.29–12 901 ppm λ‐cyhalothrin for Cyonara 9.7, 3.30–32 956 ppm imidacloprid for Dominion 2 L, 1.08–10 800 ppm acephate for Ortho Systemic Insect Killer, and 2.70 – 27 000 ppm carbaryl for Sevin Concentrate Bug Killer.

Each insecticide dilution was thoroughly mixed by vortexing briefly prior to adding to the wells. Cells were incubated in the presence of the insecticide for 24 h to assess the response of viability to acute exposure. Cells were incubated for 120 h to assess the response of viability to long‐term exposure, with readings at 0 h (baseline) and 24‐h intervals for wells assigned to the specific period. At the end of the exposure period, 10 μl of alamarBlue reagent was directly added to each well for a total volume of 110 μl. The plates were then incubated at 32 °C in the dark for 4 h from the endpoint of the exposure period prior to fluorometric analysis. Fluorometry was performed using a Cytation 5 Cell Imaging Multimode Reader connected to Gen 5 3.10 Software (BioTek). There were three experiments for both the acute and long‐term exposures, and each experiment used a separate, unique pool of cells. Each experiment consisted of six replicate wells/insecticide concentration, three to six replicate wells of untreated control cells/insecticide, and three to six wells containing 100 μl of culture medium only but exposed to the viability reagent (110 μl total volume) for blank subtraction. Wells subject to pipette errors or showing signs of contamination were few (*n* = 9/540 for acute exposure experiments; *n* = 10/1239 for long‐term exposure experiments, including all six wells of the 48‐h exposure to 0.001% Cyonara 9.7 for Experiment 2; these were excluded from analysis.

### Prediction of the LC50

To establish the predicted LC50 value of each insecticide, the viability data (% normalized to untreated control viability) from the acute response experiments were combined and plotted against the log‐transformed formulation concentration series (% v/v). The 0.0% concentration was arbitrarily changed to 0.001% and then log‐transformed to −3.0 so that it could be included in the model with the other acute response data. A four‐parameter nonlinear logistic model was then applied to the data using JMP Pro software (JMP Statistical Discovery). The data were fit with a sigmoidal curve, and the parameter estimates with 95% confidence intervals (CIs) are provided in the Supporting Information, Table 2. The custom inverse prediction tool in JMP Pro was set to 50% viability with a 0.95 confidence level to generate the predicted LC50. The predicted LC50 for each insecticide was then used as a benchmark concentration for subsequent exposure experiments to qualitatively analyze effects on cell morphology and quantify expression of a cell stress response gene.

### Influence of insecticides on cell morphology using Giemsa staining

The AmE‐711 cells were seeded at an approximate density of 1.5 × 10^6^ cells/well in a six‐well flat‐bottomed tissue culture treated plate (Corning). The cells were seeded in 2 ml of culture medium with 10% FBS and allowed to recover as described previously in the *Resazurin‐based viability assay* section. At the time of exposure, the predicted LC50 of each insecticide, as determined using the data from the acute exposure experiments, was added to each well. The total volume of medium containing the LC50 of insecticide was 1 ml. Cells were incubated in the presence of each insecticide for 12 h. Exposure to the insecticides was stopped at 12 h to limit the magnitude of cytotoxic damage. This permitted a range of morphologic changes to be present in each well, from cells having a healthy, normal phenotype to cells having a stressed, abnormal phenotype, and allowed for within‐well comparisons, as well as comparisons of insecticide‐exposed cells with untreated control cells. At the end of the 12‐h exposure period, the culture medium was discarded, and the cell layers were washed three times with 1 ml of sterile 1× dPBS/wash. The cell layers were allowed to dry for 7 min at room temperature. The cell layers were then fixed by incubation in the presence of 1 ml of absolute methanol for 9 min, with a complete change of the fixative at 3 and 6 min. The fixative was removed, and the cell layers were allowed to dry for 5 min at room temperature. The cells were then stained using 10% Giemsa Stain, Modified Solution (Millipore Sigma) in Sorenson's buffer, pH 6.8, for 60 min at room temperature. After the stain was removed, representative images were captured digitally using phase contrast microscopy with a ×60 oil immersion objective. Illumination settings (e.g., brightness/contrast, light intensity) were identical for each well at the time of image capture. Captured images were minimally processed; they were cropped, corrected for white balance, and adjusted for brightness using the auto settings of Photoshop Ver 23.1.1 (Adobe). One well containing AmE‐711 cells was exposed to each insecticide, and one well containing untreated cells was used for comparison. Kroemer et al. (2009) served as a reference to characterize changes in cell morphology.

### Quantitative reverse transcriptase polymerase chain reaction of a cellular stress response gene

We quantified expression of a cellular stress response gene to corroborate the qualitative morphological changes we observed in AmE‐711 cells undergoing cell stress and death from exposure to the predicted LC50 of each insecticide. We lengthened the duration of exposure to the predicted LC50 of each insecticide to increase the likelihood of capturing biologically relevant differences in gene expression between the insecticide‐treated and untreated control cells. The gene *lethal(2)essential for life‐like* homolog *410087a* codes for a small heat shock protein (sHsp) that was recently shown to be up‐regulated in the midgut of adult honey bees exposed to different stressors (Shih et al., 2021). For this experiment, AmE‐711 cells were seeded at an approximate density of 2.0 × 10^6^ cells/well in six‐well flat‐bottomed tissue culture treated plates and allowed to recover as described previously in the *Resazurin‐based viability assay* section. The AmE‐711 cells were exposed for 18 h to 1 ml of culture medium containing the predicted LC50 of each insecticide. Exposure was stopped at 18 h to limit cytotoxic damage, permitting recovery of sufficient quantities of high‐quality RNA for quantitative reverse transcriptase–polymerase chain reaction (RT–qPCR) analysis. The treatment medium was removed from the wells at the end of the exposure period, and the cell layers were washed three times with 1 ml of sterile 1× dPBS/wash. Total RNA was then extracted using chilled TRIzol (1 ml/well) according to the manufacturer's protocol (Invitrogen Life Technologies). The RNA was measured for quality and quantity using a Nanodrop ND‐1000 spectrophotometer (ThermoFisher Scientific), and then diluted to a concentration of 100 ng/µl with diethylpyrocarbonate‐treated, nuclease‐free water for all samples. Complementary (c)DNA was synthesized using 1 µg total RNA. Total RNA was first treated for contaminating DNA and Rnases with Dnase I and RNaseOUT at 37 °C for 30 min, followed by 75 °C for 10 min. Next, messenger (m)RNA was selected and reversed transcribed using a master mix containing oligo dT_12‐18_, dNTPs, 0.1 M DTT, and SuperScript II reverse transcriptase in 5× first strand buffer. The reaction parameters for reverse transcription were 42 °C for 50 min, followed by 70 °C for 15 min. All PCR reagents were from Invitrogen Life Technologies/ThermoFisher Scientific.

The primer sequences used for amplification of *410087a* were F 5′‐TTTCCCATTGGTGGGAAGCA‐3′ and R 5′‐AACCGTGATCATCTGCCTTGT‐3′ (Shih et al., 2021). Relative expression of the target transcript was normalized to the average of two honey bee reference genes. Primers for the reference genes β *‐actin* (*Actin*; F 5′‐TTGTATGCCAACACTGTCCTT‐3′; R 5′‐TGGCGCGATGATCTTAATTT‐3′) and *Ribosomal protein S5* (*AmRPS5*; F 5′‐AATTATTTGGTCGCTGGAATTG‐3′; R 5′‐TAACGTCCAGCAGAATGTGGTA‐3′) were previously published in Evans (2006). The RT–qPCR amplification was conducted in a reaction volume of 20 μl consisting of 10 µl of iTaq Universal SYBR Green Supermix (BioRad), 0.8 µl of each primer [10 µM], and 2 μl of cDNA template. Amplification was performed using a CFX96 Real‐Time System (BioRad) using the following temperature–time profile: 95 °C for 5 min, followed by 40 cycles of 95 °C for 5 s, 60 °C for 10 s, and 72 °C for 10 s. After the amplification step, a melt curve analysis was conducted to confirm the specificity of the PCR products, whereby the products were exposed to a temperature ramp of 55–95 °C at a rate of 0.5 °C/0.05 s for 81 cycles. There were three technical replicates for each sample, and each reaction contained a set of wells that were free of template. A biological replicate, or sample, consisted of a well containing cells that was either exposed to insecticide or was untreated. The experiment was conducted using six wells containing cells for each of the four insecticides and six wells containing untreated cells (*n* = 30). The RNA of one sample from the Sevin Concentrate Bug Killer group had to be re‐extracted due to quality issues, and PCR reactions were rerun for both reference genes and the target gene for this sample. Gene expression was calculated using the $2^{-\Delta \Delta C_{t}}$ method (Livak & Schmittgen, 2001). The proposed RNA and protein sequences for *410087a* are described in Shih et al. (2021).

### Statistical analyses

The JMP Pro 15.0.0 software (JMP Statistical Discovery) was used to make graphical summaries of the data and perform analyses when appropriate. For viability assays, the optical density value of each well exposed to insecticide was normalized to the mean optical density value of the untreated control wells. Data are reported as the mean percentage (%) viability ± standard deviation (per experiment) or standard error (experiments combined) per insecticide concentration for acute and long‐term exposure experiments, respectively. A one‐way analysis of variance (ANOVA) was used to test the fixed effect of treatment on relative expression of *410087a*. A Dunnett's test was used for post hoc comparisons of the mean fold‐change in relative expression of *410087a* for each treatment group compared with the untreated control. Data from the long‐term exposure assay were analyzed using a mixed model ANOVA with concentration (0.0%, 0.001%, and 0.01%) and time of exposure (0, 24, 48, 72, 96, and 120 h) treated as fixed effects and experiment treated as a random effect. If a significant interaction was found, a Tukey's Honest Significant Difference test was used for post hoc comparisons of the treatment group with the untreated control at each time point.

## RESULTS

### Response of AmE‐711 cell viability to acute insecticide exposure

**Figure 2:**
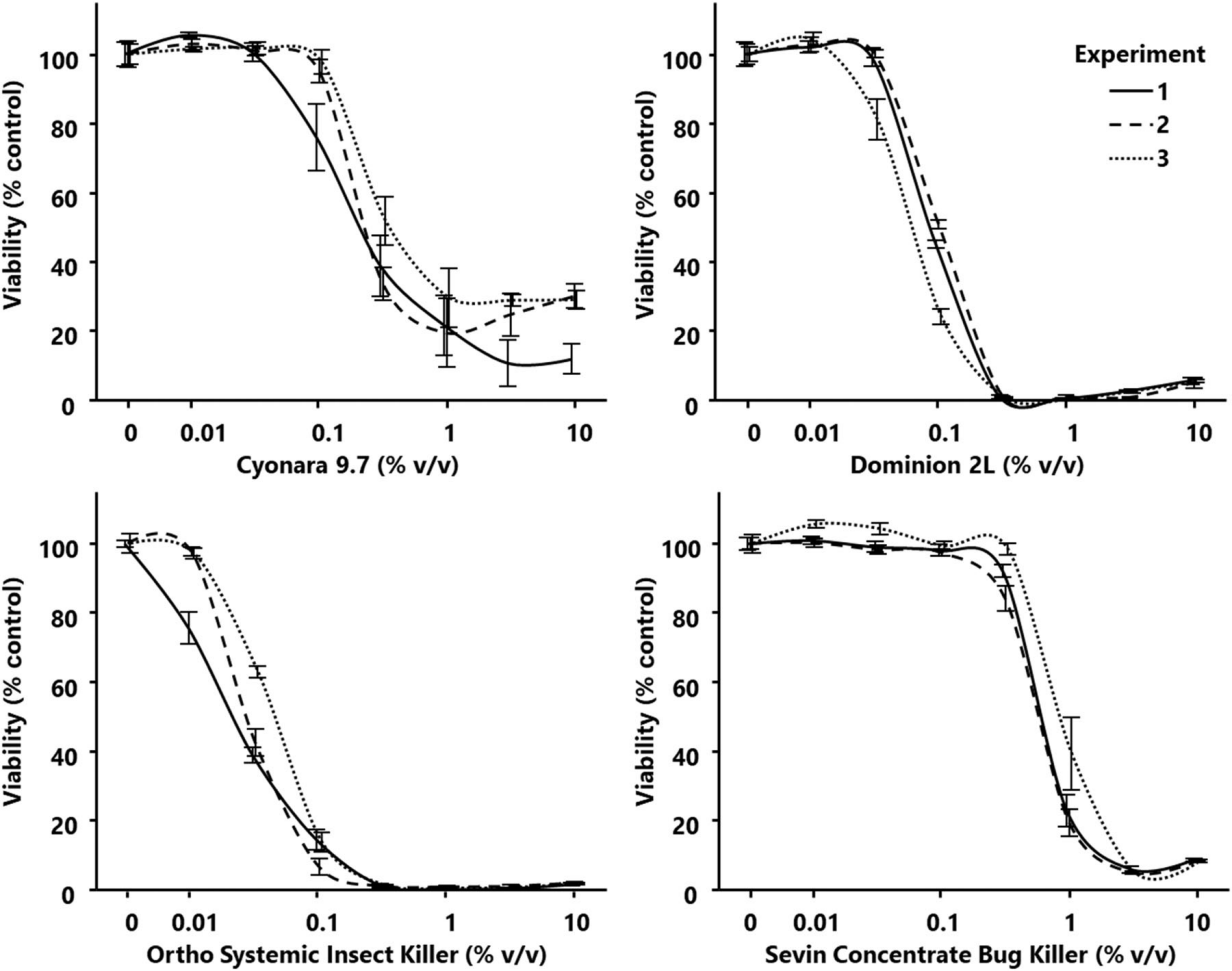
The response of AmE‐711 cell viability to four formulations each containing a different insecticide. The AmE‐711 cells were incubated in the presence of different concentrations of each formulation (0.01, 0.05, 0.1, 0.5, 1.0, 5.0, and 10.0% v/v) for 24 h, followed by a fluorescent resazurin‐based assay to establish cell viability. Optical density data are presented as the mean viability (%) normalized to the untreated control (0.0%) ± standard deviation for each of three experiments.

We evaluated the response of AmE‐711 cell viability to an acute exposure of formulations containing a pyrethroid (λ‐cyhalothrin), a neonicotinoid (imidacloprid), an organophosphate (acephate), and a carbamate (carbaryl). Figure 2 depicts the change in AmE‐711 cell viability after exposure to a dilution series of each formulation for 24 h. The AmE‐711 cells showed a concentration‐dependent reduction in viability within the range of formulation concentrations tested (0.0%, 0.01%, 0.05%, 0.1%, 0.5%, 1.0%, 5.0%, and 10.0%). Cell viability had decreased to at or near 0% for formulation concentrations of 0.5% or higher for Dominion 2 L (1648 ppm or more of imidacloprid) and Ortho Systemic Insect Killer (540 ppm or more of acephate) and 5.0% or higher for Sevin Concentrate Bug Killer (13 500 ppm or more of carbaryl). Viability did not reach 0% for cells exposed to any tested concentration containing Cyonara 9.7, but levels did stabilize and remain relatively unchanged at 1.0% or higher of the formulation (1290 ppm or more of λ‐cyhalothrin). Visual inspection of wells containing cells exposed to 1.0% or more of Cyonara 9.7 showed no difference in alamarBlue dye coloration compared with blanks; therefore, we conclude that the viability of cells exposed to 1.0% or more of Cyonara 9.7 was at or near 0%.

### The predicted LC50 for each insecticide

Having demonstrated a concentration‐dependent reduction in cell viability after 24‐h exposure to each formulation, we next wanted to use the acute exposure data to predict the LC50 for each formulation and its corresponding active ingredient. The viability data from the three acute exposure experiments were combined and used to construct a four‐parameter logistical model for each formulation. The data were then fit with a sigmoidal curve; the slope and other parameter estimates with 95% CIs specific to each model are presented in Table 2 and the Supporting Information, Table 2, respectively. The goodness of fit (*R* ^2^) was 0.95 or more for each model. The models were then used to predict the LC50 for each formulation using the JMP Pro software. The predicted LC50 (log_10_) with 95% CI was −0.45 (−0.50, −0.39) for Cyonara 9.7, −1.04 (−1.05, −1.03) for Dominion 2 L, −1.31 (−1.33, −1.30) for Ortho Systemic Insect Killer, and −0.10 (−0.11, −0.08) for Sevin Concentrate Bug Killer. Knowledge of the concentration of active ingredient listed on the product label and the dilution scheme used for the acute exposure experiments allowed us to transform the LC50 from a log_10_ value to ppm of active ingredient for each formulation. The transformed predicted LC50 with 95% CI was 461 ppm (409, 521) for λ‐cyhalothrin, 301 ppm (293, 309) for imidacloprid, 52 ppm (50, 55) for acephate, and 2166 ppm (2106, 2228) for carbaryl.

**Table 2:** Estimated half‐maximal lethal concentration (LC50) for each insecticide

Formulation	Active ingredient	Slope (95% CI)^a^	R^2^ b	log_10_LC50 (95% CI)^c^	LC50 ppm (95% CI)^d^
Cynoara 9.7	λ‐cyhalothrin	−4.38 (−5.13, −3.62)	0.95	−0.45 (−0.50, −0.39)	461 (409, 521)
Dominion 2 L	Imidacloprid	−9.43 (−10.84, −8.01)	0.98	−1.04 (−1.05, −1.03)	301 (293, 309)
Ortho Systemic Insect Killer	Acephate	−6.69 (−7.81, −5.57)	0.97	−1.31 (−1.33, −1.30)	52 (50, 55)
Sevin Concentrate Bug Killer	Carbaryl	−11.81 (−13.01, −10.62)	0.99	−0.10 (−0.11, −0.08)	2166 (2106, 2228)

^a^ Data from three acute toxicity experiments were used to construct a four‐parameter nonlinear logistic model to obtain the slope with 95% confidence interval. Additional parameter estimates are in the Supporting Information, Table 1.

^b^ The goodness of fit for the four‐parameter nonlinear logistic model.

^c^ The JMP Pro custom inverse prediction tool was used to estimate the log_10_LC50 with 95% CI.

^d^ The log_10_LC50 value was transformed to the antilog (% w/v) and then converted to the estimated parts per million (ppm) based on the concentration of active ingredient reported on the formulation label.

CI = confidence interval.

### Morphological changes in AmE‐711 cells after 12‐h exposure to the predicted LC50

**Figure 3:**
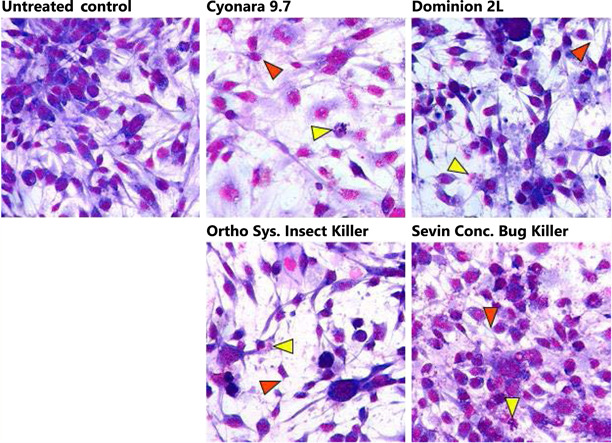
Changes in the morphology of AmE‐711 cells exposed for 12 h to the predicted half‐maximal lethal concentration of four formulations each containing a different insecticide. Untreated control cells have a centrally located nucleus (magenta) and are supported by a spindle‐shaped cytoplasm (violet). Cells exposed to the formulations show thinning or retraction of the cytoplasm and a corresponding reduction in cell volume (orange arrowhead). Cells exposed to the formulations also show loss of plasma membrane integrity and fragmentation of the nucleus (yellow arrowhead).

We used the predicted LC50 values as the basis for exposing AmE‐711 cells to the insecticides so we could observe changes in cell morphology that would suggest cell stress and death (Figure 3). Healthy, untreated AmE‐711 cells stained with Giemsa presented a typical fibroblast‐like morphology consisting of a centrally located nucleus and spindle‐shaped cytoplasm. The morphology of cells exposed to each insecticide deviated from the control condition. The spindle‐like cytoplasm, extending linearly from the nuclear area in healthy cells, was lost or retracted in some exposed cells, regardless of type of insecticide. In addition to this rounding‐up formation, the nucleus of some insecticide‐exposed cells was condensed or fragmented, which was accompanied either by destruction of the plasma membrane or occurrence of membrane‐bound bodies.

### Relative expression of *410087a* in AmE‐711 cells exposed to the predicted LC50

**Figure 4:**
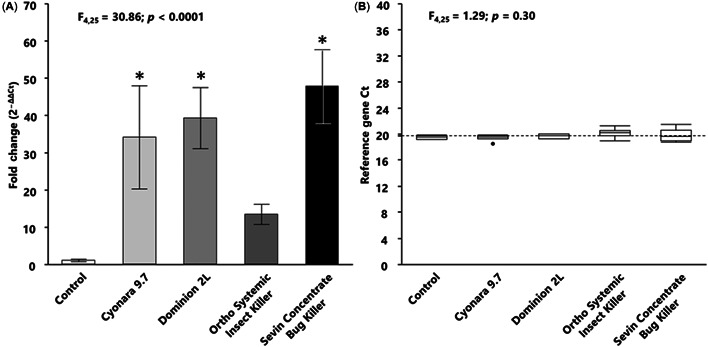
Induction of a honey bee cellular stress response gene in AmE‐711 cells exposed to four formulations each containing a different insecticide (*x*‐axis). (**A**) Fold induction of the small heat shock protein *410087a* in cells exposed for 18 h to either the predicted LC50 of each formulation or untreated culture medium. Data are summarized as the mean fold induction ± standard deviation and were calculated using the $2^{-\Delta \Delta C_{t}}$ method. There was a significant effect of treatment on *410087a* expression (*F*_4,25_ = 30.88; *p* < 0.0001). A Dunnett's test was used to make multiple comparisons with the untreated control group; *denotes a significant difference from the untreated control group. (**B**) The reference gene *C*_t_ values were calculated using the average *C*_t_ values of two honey bee reference genes, *Actin* and *AmRPS5*. Reference gene data were similar regardless of treatment (*F*_4,25_ = 1.29; *p* = 0.30). The dashed line marks the overall mean reference gene *C*_t_ value. *p* < 0.05 was considered statistically significant.

We next examined the expression of a cellular stress response gene to add support to our microscopic observations of AmE‐711 cells undergoing cell stress and death in the presence of insecticides. We chose to quantify the relative expression of *410087a*, a sHsp shown in honey bees to be responsive to several forms of stress, including thermal and chemical stress (Shih et al., 2021). Exposure of AmE‐711 cells to the predicted LC50 of each insecticide for 18 h had a significant effect on the relative expression of *410087a* (*F*_4,25_ = 30.86; *p* < 0.0001; Figure 4A). Compared with the control group, the average fold difference in relative *410087a* expression was 33.04 for Cyonara 9.7 (least significant difference [LSD] = 20.21; *p* < 0.0001), 38.23 for Dominion 2 L (LSD = 25.41; *p* < 0.0001), and 46.82 for Sevin Concentrate Bug Killer (LSD = 34.00; *p* < 0.0001). Expression of *410087a* in cells exposed to Ortho Systemic Insect Killer was also increased (by 12.41‐fold) compared with control cells but this difference was not statistically significant (LSD = −0.42; *p* = 0.06). The mean reference gene *C*_t_ values, which were calculated using the average *C*_t_ values of two honey bee reference genes, *Actin* and *AmRPS5*, were similar regardless of treatment (*F*_4,25_ = 1.29; *p* = 0.30); this stable expression provided validation for their use to normalize the *410087a* data (Figure 4B).

### Effects of long‐term exposure to low concentrations of insecticide on AmE‐711 viability

**Figure 5:**
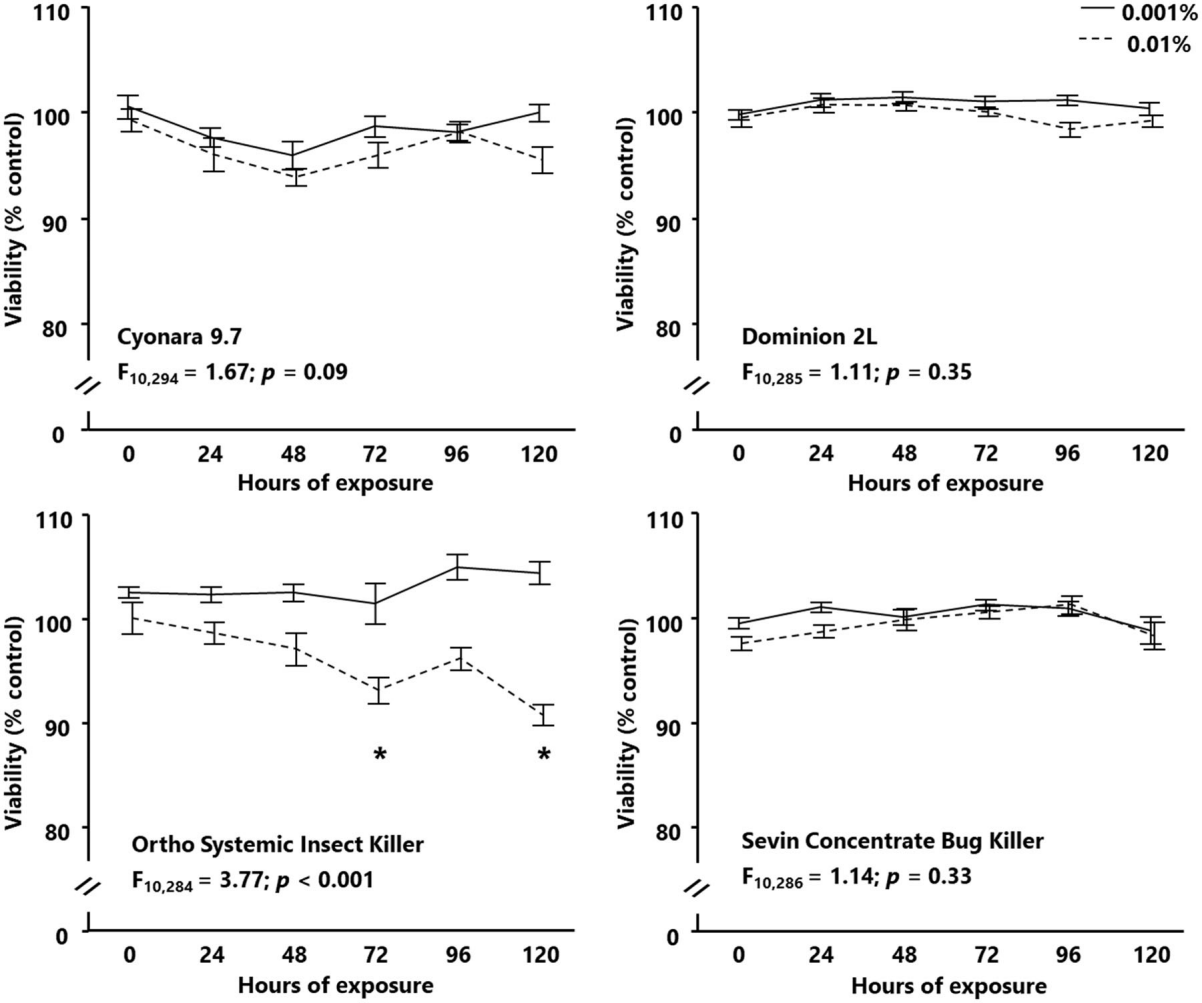
The long‐term response of AmE‐711 cell viability to sublethal concentrations of four formulations each containing a different insecticide. The AmE‐711 cells were exposed for 120 h to 0.001% and 0.01% (v/v) of each insecticide. Cell viability was measured at 0‐h and 24‐h intervals after the start of insecticide exposure using a fluorescent resazurin‐based assay. Optical density data are presented as the mean viability (%) of three trials normalized to the untreated control (0.0%) ± standard error. Data were analyzed using a mixed model analysis of variance with concentration and exposure time as fixed effects and trial as a random effect; *denotes a significant difference from the untreated control group/time point for 0.01%. *p* < 0.05 was considered statistically significant.

The effects of long‐term exposure to low concentrations of insecticide on AmE‐711 viability were limited and formulation specific (Figure 5). Of the four insecticides tested, the only significant interaction between insecticide concentration and time of exposure was for cells exposed to Ortho Systemic Insect Killer (*F*_10,284_ = 3.77; *p* < 0.0001). The viability of cells exposed to 0.01% Ortho Systemic Insect Killer (11 ppm acephate) for 72 and 120 h was significantly different from untreated cells, but 0.001% Ortho Systemic Insect Killer (1.1 ppm acephate) was not different from untreated cells at any time point. The viability of cells exposed to 0.001% and 0.01% was not different compared with untreated cells for Cyonara 9.7 (1.3 and 13 ppm λ‐cyhalothrin; *F*_10,294_ = 1.67; *p* = 0.09), Dominion 2 L (3.3 and 33 ppm imidacloprid; *F*_10,285_ = 1.11; *p* = 0.35) and Sevin Concentrate Bug Killer (2.7 and 27 ppm carbaryl; *F*_10,286_ = 1.14; *p* = 0.33). The random effect, experiment, was not significant for each insecticide (*p* ≥ 0.34).

## DISCUSSION

Due to the many opportunities for honey bees to become exposed to insecticides, it is important to expand our knowledge of their toxic effects at not only the individual bee and colony levels, but also the cellular level using in vitro models. We demonstrate that the AmE‐711 honey bee cell line can serve as a platform for in vitro toxicity testing to inform about the effects of insecticides on honey bee health. The AmE‐71l cell line showed sensitivity to four formulations, each containing a different insecticide as the active ingredient. We observed a concentration‐dependent reduction in cell viability after a 24‐h exposure and found limited evidence for a time‐dependent effect of sublethal concentrations for the four insecticides tested. Furthermore, AmE‐711 cells exposed for 12 h to the predicted LC50 of each insecticide showed hallmarks of cell stress and death based on changes in cell morphology. These morphological changes were corroborated by induction of the sHsp gene, *410087a*, a homolog of the *lethal(2)essential for life‐like* gene whose expression in honey bees has previously been found to be sensitive to various stressors (Shih et al., 2021).

The commercial products used for the present study were not chosen to make a comparative assessment or convey the potential risks of the specific formulations or their active ingredients on honey bee health, but rather to demonstrate the responsiveness of AmE‐711 cells to real‐world insecticide exposure. Application of the formulations to AmE‐711 cells was not without challenges. Some of the formulations we tested are supplied either as a suspension (Cyonara 9.7, Ortho Systemic Insect Killer, and Sevin Concentrate Bug Killer) or an emulsion (Dominion 2 L). Due to the physical properties of these formulations, it was difficult to maintain a uniform distribution of the insecticides in the culture medium. This lack of uniformity may have led to variability between the acute response patterns, as was seen for the three experiments for Ortho Systemic Insect Killer. Moreover, the turbidity of the formulations may have interfered with detection of the fluorescent signal. We did note some signal interference when the formulations were analyzed in the absence of cells at concentrations of 5.0% or more. This artifact was mitigated when cells were added to the culture environment and is supported by the typical sigmoidal response curves of the acute exposure experiments (Figure 2), as well as consistent changes in cell morphology (Figure 3) and induction of *410087a* (Figure 4) in the LC50 exposure experiments. Signal interference at high concentrations may also explain the slight, yet noticeable inflation of viability for cells exposed to 10.0% Dominion 2 L (32 956 ppm imidacloprid) and Sevin Concentrate Bug Killer (27 000 ppm carbaryl), as well as stagnation of viability above 10% for cells exposed to 5.0% or more Cyonara 9.7 (12 901 ppm λ‐cyhalothrin). Some studies reporting on the toxicity of formulations in vitro use dimethyl sulfoxide (DMSO) as a vehicle (Defarge et al., 2016; Mesnage et al., 2014). The use of DMSO likely increased formulation solubility in these studies. We did not use DMSO or similar solvent to improve solubility, but we recommend their inclusion moving forward because it may resolve issues of signal interference with fluorescence‐based detection assays.

Although working with formulated products in a cell culture system presented technical challenges, we were able to predict the LC50 for each insecticide. This prompts the question: How well do the predicted LC50 values from our acute exposure experiments using a honey bee cell line compare with lethality data from experiments in which whole bees are exposed? For example, our predicted LC50 for λ‐cyhalothrin (Cyonara 9.7) was 461 ppm in AmE‐711 cells after a 24‐h exposure, which lies between the LC50 values reported for young adult worker bees exposed for 24 h (139 ppm; Farooqi et al., 2019) and 48 h (502 ppm; Wang et al., 2020; 575 ppm; Zhu et al., 2015). For imidacloprid (Dominion 2 L), our predicted LC50 was 301 ppm, which again lies within the range of LC50 values reported for honey bee larvae exposed for 72 h (139 ppm; Dai et al., 2017) and young adult worker bees exposed for 48 h (552 ppm; Zhu et al., 2015). Our predicted LC50 for acephate (Ortho Systemic Insect Killer) was 52 ppm, which is below values reported for young adult worker bees exposed for 24 h (84 ppm; Farooqi et al., 2019) and 48 h (126 ppm; Zhu et al., 2015). These values are relatively lower compared with the other insecticides we tested but may support the possibility that honey bees are generally sensitive to acephate. On the other hand, of the four formulations we tested, we found that AmE‐711 cells were least sensitive to carbaryl (Sevin Concentrate Bug Killer), which had a predicted LC50 of 2166 ppm. The reported LC50 values for carbaryl in honey bees are variable, ranging from 44 ppm in larvae exposed for 72 h (Yang et al., 2020) to 895 ppm for young adult workers exposed for 48 h. We conclude that results obtained from toxicity testing conducted in vitro using the AmE‐711 cell line can align with those from whole‐bee studies, but we caution that consideration should be given to differences in exposure time, method of insecticide administration, and life stage tested when making these comparisons. We also emphasize that we did not test the active ingredients in their pure form; therefore, our reporting of the LC50 values in ppm of active ingredient is based on an approximation of its concentration and does not consider other factors inherent to the formulations that may have contributed to the overall toxicity. Further studies that are a combination of toxicity testing and molecular or biochemical measurements will add depth to how well the response of AmE‐711 cells to insecticide exposure correlates with higher levels of honey bee organization.

Exposure of AmE‐711 cells for 18 h to the predicted LC50 of each insecticide resulted in induction of *410087a* expression. This up‐regulation of a gene coding for a cellular stress response protein provides evidence that: 1) molecular processes of AmE‐711 cells are responsive to stressors, including different groups/subgroups of insecticides; 2) insecticide‐induced changes in these processes are quantifiable; and 3) these changes can be applied to the characterization of honey bee cell dysfunction and toxicity caused by insecticides under real‐world scenarios. The sHsps like *410087a* are phylogenetically conserved chaperones that are responsive to stressful conditions within the cell environment and act to correct protein folding/refolding and guide transport and degradation of new and damaged proteins (Boopathy et al., 2022). Induction of sHsps to insecticide exposure provides insight into the regulatory networks governing the honey bee cellular stress response (Shih et al., 2021). Future experiments using the AmE‐711 cell line could incorporate the use of bioinformatics, coupled with functional assays, to resolve mechanisms of cell death, or tolerance, as it relates to insecticide‐induced activation of the cellular stress response network. Also, because the insecticides used in our study target neuronal receptors and enzymes (Insecticide Resistance Action Committee, 2022), an added component of future experiments should explore whether AmE‐711 cells possess neuronal properties. This could involve determining the presence and functionality of receptors or enzymes specific to neurons and neuromuscular junctions, as well as identifying secondary targets (Xu et al., 2022). Broader resolution of the effects on cell signaling pathways and other biochemical processes could lead to the development of insecticides that have reduced negative outcomes for honey bees. Moreover, it could help firm linkages between the proximate interaction of a toxin with a target molecule and the ultimate outcome of honey bee colony failure (i.e., an adverse outcome pathway; Christen et al., 2018; LaLone et al., 2017).

Honey bees are frequently exposed to a combination of abiotic and biotic stressors simultaneously. Interactions among these stressors could accelerate the disease state and cause premature death of affected individuals or colonies. Two stressors that have received increased attention due to their potential to interact and negatively affect honey bee health are insecticides and viruses. Honey bees co‐exposed to sublethal doses of insecticides and viruses have greater viral titers than bees infected with viruses alone (Di Prisco et al., 2013; Doublet et al., 2015). We were able to show that AmE‐711 cells had limited to no reduction in viability when exposed to sublethal concentrations of each insecticide for 120 h. This lack of an effect sets the stage for continuing experiments using AmE‐711 as a model to explore how sublethal insecticide exposure, similar to what honey bees may experience in the hive, perturbs cell structure, and how such exposure functions to promote virus infection and replication (Di Prisco et al., 2013; Parekh et al., 2021).

Concurrent exposure to different insecticides is another interaction that is of concern for honey bee health (Mullin et al., 2015). Two or more insecticide formulations are frequently mixed and then applied in a single application (i.e., tank mixing). Tank mixing is convenient and can improve pest control, but these mixtures, which likely include adjuvants, may result in additive or synergistic toxicity for honey bees (Wade et al., 2019; Walker et al., 2022). Furthermore, formulations are themselves a blend of active and inert ingredients. Although active ingredients are typically the focus of toxicity studies, inert materials, whether alone or as part of a formulation or tank mixture, have been shown to produce lethal and sublethal effects (Chen et al., 2019; Mullin et al., 2016). We too assumed that the active ingredients were the drivers of the toxicity we observed, but we cannot rule out that the inert ingredients in the products we used may have contributed to these effects. Our understanding of the toxicity of inert ingredients for bees has not kept pace with the prevalence of their use in formulations and adjuvants (Straw et al., 2022). Complicating this issue is that the identity of inert ingredients used in formulations is often unknown (Straw et al., 2022). Research rooted in the use of cell lines could provide a convenient, cost‐effective platform to help fill knowledge gaps pertaining to inert ingredients. For example, AmE‐711 could be used in screening arrays in which cellular, molecular, and biochemical outputs facilitate partitioning the toxicity of individual and combinations of technical‐grade compounds, inert materials, formulations, and adjuvants.

## CONCLUSIONS

The AmE‐711 cell line was established from honey bee embryonic tissues in 2011 (Goblirsch et al., 2013) and is still in its infancy as a research tool for honey bee biology and disease. We wanted to assess the utility of AmE‐711 as a research tool in one area of honey bee biology and disease, insecticide toxicology. Exposure of AmE‐711 cells to formulations containing different groups of insecticides resulted in a concentration‐dependent loss of cell viability, and also led to atypical changes in cell morphology and up‐regulation of a sHsp gene associated with the cellular stress response. The ability to characterize the response of AmE‐711 cells to different groups of insecticides, both quantitatively and qualitatively, means AmE‐711 could be added as a complement to whole‐organism or colony‐level testing, especially early in the screening process of new insecticides or the re‐evaluation of existing insecticides (Fischer et al., 2019; Smagghe & Swevers, 2013). Ultimately, AmE‐711 could serve as a research and hazard assessment tool to minimize the risks and maximize the benefits that insecticides offer for honey bees and the environment.

## Supporting Information

The Supporting Information is available on the Wiley Online Library at https:/10.1002/etc.5500.

## Conflict of Interest

The authors declare no conflict of interest.

## Disclaimer

Reference herein to any specific commercial products, process, or service by trade name, trademark, manufacturer, or otherwise, does not necessarily constitute or imply its endorsement, recommendation, or favoring by the United States Government. The views and opinions of authors expressed herein do not necessarily state or reflect those of the United States Government, and shall not be used for advertising or product endorsement purposes.

## Author Contributions Statement

**Michael Goblirsch**: Conceptualization; Data curation; Formal analysis; Investigation; Methodology; Project administration; Resources; Visualization; Writing—original draft and review & editing. **John J. Adamczyk**: Conceptualization; Funding acquisition; Investigation; Methodology; Project administration; Resources; Supervision; Validation; Visualization; Writing—review & editing.

## Supporting information

This article includes online‐only Supporting Information.

Supporting Tables.

Supporting Acute.

Supporting Chronic.

Supporting GeneExpressionAnalysis.

## Data Availability

The data for the present study are available as the Supporting Information.
